# Between the Urgent and the Emerging: Representations on Sex Education in the Debate for Abortion Legalization in Argentina

**DOI:** 10.3389/fsoc.2021.635137

**Published:** 2021-06-07

**Authors:** Gabriel Dvoskin

**Affiliations:** Linguistic Department, University of Buenos Aires, Buenos Aires, Argentina

**Keywords:** sex education, abortion, parliamentary debate, discourse analysis, Ni Una Menos

## Abstract

The aim of this article is to analyse, within the framework of discourse analysis, the social representations that circulated about the sex education in the parliamentary debate on the bill for the Voluntary Interruption of Pregnancy that took place in the Chamber of Deputies, on June 13, 2018, in Argentina. To do this, we review the functions attributed to sex education by both legislators who voted in favor of the bill and those who voted against. In turn, we analyze the topics that were related to this issue, as well as the social actors involved and those who were legitimized to address the problem. We aim to establish if the feminist discourse that was constituted and consolidated in Argentina from the *Ni Una Menos* march, in 2015, managed to impose its agenda on the political sphere. Specifically, we are interested in investigating whether the representations that were put into circulation about sex education were retaken in the parliamentary debate on the voluntary termination of pregnancy bill, a space in which sex education constituted a preponderant topic.

## Introduction

In the year 2018, a historical event took place in Argentina: the bill for the Voluntary Interruption of Pregnancy (*IVE* by its Spanish initials^1^) was debated for the first time in the National Congress. Although the bill had already been introduced by the National Campaign for the Right to Legal, Safe, and Free Abortion (henceforth *the Campaign*) on seven occasions, it had never before received sufficient support from legislators to be debated in the parliamentary chamber. Unlike previous years, the support for the bill by 71 deputies and the subsequent authorization of its discussion by then President Mauricio Macri (2015–2019) allowed the debate for the legalization of abortion to be included on the parliamentary agenda of that year.

As a result, the matter circulated widely at the social level in Argentina, not only in the political and media spheres, but also in educational and religious institutions -mainly Catholic and Evangelical- and even in the intimate family realm (Felitti and Prieto, 2018). In fact, the issue of abortion was addressed in spaces where it had never before been dealt with, both in the public and private domains, ranging from major television programs to family and friend gatherings. Different people, performing very diverse social roles and from a wide range of political and ideological orientations, felt they ought to express their position on an issue that, until that moment, had remained undisclosed for the vast majority of society (Felitti and Ramírez Morales, 2020).

The massive march called “Ni Una Menos” (Not One Less)^2^, in June 2015, changed the situation in Argentina in the matter of gender (Faur, 2020). This new situation inspired different feminist movements, grouped under the Campaign that originated in 2005^3^, who managed to have the content of the IVE bill addressed, in the first place, in a plenary of commissions of the Lower House of Congress. In the course of 2 months, 15 sessions were held in the Health, Family and Criminal Legislation Committees, in which 738 representatives from different disciplines defended their positions from a large variety of perspectives.

On June 13, 2018, the bill obtained the majority of votes in the Lower House of Congress, with 129 votes in favor, 125 against and one abstention. However, almost 2 months later, on August 8, it was rejected in the Senate, with 38 votes against the bill, 31 in favor of it, two abstentions and one absence. Despite the defeat, the treatment of the bill in parliament and the wide circulation of the issue at the social level made a series of problems visible that strictly exceed those related to abortion. Thus, this bill could be included within a larger set of measures that have been passed in Argentina since 2005 and that tackle gender and sexuality matters (Báez and Fainsod, 2018)^4^.

The slogan formulated by the Campaign for the promotion of the debate was “Sex education to decide, contraceptives not to abort, legal abortion not to die,” which puts the importance of sex education in schools in the spotlight. Paradoxically, this argument was also taken up by groups and sectors of society that opposed the passing of the bill (Dvoskin and Estivalet, 2020), which makes it clear that, beyond the fact that the Comprehensive Sex Education (*ESI* by its Spanish initials^5^) law was passed in 2006, in Argentina there still exists a dispute over what functions such law should fulfill, what modalities it should adopt, and what effects its implementation is expected to generate.

In this article, we analyze, by means Discourse Analysis tools, the representations that circulated about sex education in the debate on the IVE bill that took place in the Lower House of Congress, on June 13, 2018. To do this, we look at the functions attributed to sex education by both legislators who voted in favor of the IVE bill and those who voted against it. In turn, we analyze the topics that were related to this issue, as well as the practices and social actors involved and those who were constituted as legitimate speakers to address the matter.

Therefore, we aim, on the one hand, to investigate which are the different discourses on sex education that have coexisted in Argentina since the change of socio-historical conjuncture established after the “Ni Una Menos” march in 2015. We are particularly interested in addressing the disputes that have arisen around this issue, as well as the points of agreement among the different sectors. In addition, based on this analysis, we problematize what regularities remain and what changes have taken place in the representations on sex education in Argentina since the passing of the law in 2006, through the debate about the IVE bill in 2018.

The general objective of the article is to determine if the feminist discourse^6^ that is consolidated in Argentina from 2015 onward managed to establish its agenda in the political sphere. Specifically, we are interested in investigating whether the topics that were put into circulation at the social level in relation to ESI and the signs and evaluations that were used to address them were taken up again in the parliamentary debate on the IVE bill, a space in which sex education constituted a key theme.

We have organized the article into four sections. In the first one, we present the theoretical assumptions and the methodological tools that guide our research. In the second section, we characterize, on the one hand, the historical, social and political conjuncture in which the ESI law was enacted in 2006, and we mention the controversies that were created around its implementation at the first stage. On the other hand, in this same section, we describe the subsequent situation established by the *Ni Una Menos* march, in 2015, which broadened the panorama in matters pertaining to gender and sexuality on the public agenda and in relation to ESI. Once the different conjunctures have been characterized, in the third section we present the analysis of the representations that circulated on ESI in the parliamentary debate about IVE. We reserve the final section of the article for the discussion of the results obtained from the analysis and for our final remarks.

## Materials and Methods

Our research emerges from considering discourse analysis as a way to access social analyses (Raiter, 2008). This assumption is derived from conceiving the use of language as a social practice (Fairclough, 1992), a characteristic that presents language use not only as a reflection of the world it names, but also as a constitutive element of that reality, to which it grants values and meaning. This property is evidenced by the fact that every political regime, economic measure or social movement needs to be accompanied by a discourse that sustains and legitimizes it (Angenot, 1989), which is why discourse constitutes a tool for the reproduction of the social order, but also for its questioning and transformation.

Its analysis demands, therefore, to take into account the socio-historical conditions in which discourses circulate, which include factors that go beyond the strictly linguistic aspects and involve knowledge of other social disciplines, such as History, Philosophy, Sociology, Anthropology, Politics, Psychology or Economics, a fact that makes Discourse Analysis an eminently interdisciplinary field. The intersection between Linguistics and the rest of the Social Sciences has as a meeting point the semantic dimension of discourses, which entails the focus of this type of research.

Addressing the question of meaning obliges us, as analysts, to leave aside the intentions and wills of the speaking subject and to focus instead on the effects that the text generates (Verón, 1986), which do not constitute an inherent property of the text as a product, but rather of the types of relations (solidarity, complementarity, confrontation, rejection) that it establishes with the rest of the discourses that circulate in a given social formation (Pêcheux, 1975). It is the universe of the sayable, the discursive network, which accords value to the signs and expressions that appear in a specific text, so we must problematize how the text is inserted in that network. Analyzing the realm of possible meaning effects of a given text allows us to observe which ideological contents circulate in a society, which are the discourses that, with greater or lesser preeminence, are enabled and define the historical limits of the sayable and the thinkable (Angenot, 1989).

Now, a text generates, in parallel fashion, a set of interpretations that are not possible: there is a whole series of meanings that cannot be attributed to texts, either because they are considered absurd, utopian, humorous, or because they place them on the plane of the unthinkable or the impossible. Both the possible and the not possible interpretations are established by their reference to the *dominant discourse* (Raiter, 1999), an axis that includes the most widespread, most accepted meanings of a given sign or expression. It is from the dominant discourse that qualifications are made possible, that value is attached to the rest of the discourses circulating on the network: statements are read, heard, and interpreted according to the distance they maintain from the dominant discourse. This makes it unnecessary to exclude or censor certain discourses, for these discourses will be deemed (true, fictional, marginal, pornographic, etc.) by their reference to the dominant discourse.

From this approach, there is a pre-existing referentiality to every individual text, which establishes not only the values of the signs that appear in it, but also legitimizes what issues it can address, what can be debated, what is controversial, what can be said and who can do so. This discursive initiative gives the dominant discourse the power to contain (topics, signs, values), while the other discourses will limit themselves to responding: beyond that which they deny, reject or criticize, the other discourses will constitute themselves *as opposing discourses* to the dominant discourse (Raiter, 1999), so that they will be integrated into the latter, confirming, and reinforcing it.

A discourse that seeks to leave this merely dissenting role must constitute itself as a discourse opposing the dominant discourse: questioning its axis of references, criticizing the values imposed by it and imposing new signs and topics of discussion. Only in this way will the dominant discourse be unable to qualify it and will be forced to respond to it, a fact that will make it lose its discursive initiative. A discourse that achieves this type of relationship would constitute itself as an *emerging discourse* (Raiter, 1999), defined as one that changes the existing references and then forms a new dominant discourse.

In this article, we investigate whether the feminist discourse that was put into circulation in Argentina, as of 2015, was constituted as an emerging discourse, that is, whether it managed to impose the topics of debate and the signs and assessments with which to deal with issues related to gender and sexuality. Or if, on the contrary, it was permeated by the axis of references already established by the dominant discourse, so that it was configured as an opposing discourse within the prevailing order. We focus our research specifically on the representations built around sex education, a topic that has become a subject of controversy especially since its sanction as a national law in 2006 (Dvoskin, 2015), and whose implementation has been put forward as an argument not only by those who supported the IVE bill but also by those who were against it, a paradox that highlights the dispute that revolves around this measure.

To that end, we have analyzed the deputies' presentations that problematized the implementation of ESI at the debate over the IVE bill by establishing a thematic path (Zoppi Fontana, 2005) defined by the appearance of the ideological sign (Voloshinov, 1929) “sex education,” which allowed us to delimit the corpus with which to work. The methodology employed is of a qualitative type, given that what interests us is the characterization of the different discourses—with their own signs and evaluations—that were put into circulation in relation to ESI in the parliamentary debate. Beyond the vote for or against the bill or the predominance of some discourses over others throughout the presentations, our research is centered on reconstructing the argumentative logics (Angenot, 2015) that were mobilized around ESI. That is, we are interested in revealing what contents appear to be formulated as premises on this topic—and are, therefore, exempt from any kind of debate or questioning; and what are the more or less explicit *topoi* or discursive guarantors (Ducrot, 1988) that allow for the enchainment that lead to attributing certain meanings and functions to sex education.

We propose the concept *discursive scene* (Dvoskin, 2017) as an entry point for approaching the texts, which refers to the configured identities of the participants in a given conjuncture and of the discursive positions evoked, including those of the speaker (Ducrot, 1984) and those of the addressee(s). Thus, we analyze the images that the deputies construct for themselves and the social roles from which they position themselves to develop their arguments, what practices they associate with sex education, what social actors they connect with these practices and what roles they play in the actions they mention, from what linguistic resources they bring to the fore the voices they include and what attitudes they adopt in the face of the various positions they evoke.

The analysis of the discursive scene configured in the IVE parliamentary debate in relation to sex education allows us, on the one hand, to establish which are the different discourses that coexist in Argentina today with respect to ESI: which are the points of agreement on the subject and in which axes the controversy resides. And, on the other hand, to determine the regularities and ruptures that it presents with respect to the scene constituted on this topic in the situation prior to 2015. Thus, we will problematize the extent to which the discourse promoted by the feminist movements at a social level managed to be inscribed in the political sphere.

## A Brief Overview of ESI

In this section, we will briefly characterize the path taken by ESI in Argentina, from its sanction as a law, in 2006, to the IVE debate, in 2018. We follow Faur (2020) in the division of this trajectory into two stages according to two discursive conjunctures (Choulariaky and Fairclough, 1999): the sanction of ESI as a national law in 2006, and the “Ni Una Menos” march in June 2015.

The first stage that we will characterize begins in 2006, with the sanction of the law, which marked a break from previous experiences in the field of sex education (Faur, 2012) and includes the period of ESI institutionalization, which covers the curricular guidelines established in 2008 and the modalities adopted for its implementation at an initial moment. The second stage, on the other hand, begins with the situation that was inaugurated with the “Ni Una Menos” march, in June 2015, which broadened the agenda on gender and sexuality at a social level and in relation to ESI in particular (Morgade and Fainsod, 2019), by putting into circulation new topics and new perspectives from which to approach them and by bringing to the fore the voices of social actors that until that moment had been silenced or had had a marginal status.

### The Institutionalization of ESI—Comprehensive Sex Education

On October 4, 2006, Argentina passed National Law 26,150, known as the “Comprehensive Sex Education Law,” *ESI* by its Spanish initials, which made it compulsory to provide sex education in all schools in the country, both state-run and privately-run, from the initial to higher levels of teacher training and non-University technical education. The broad consensus that was reached in parliament (with only one vote against in each of the Houses) was mainly because the bill was presented in response to a series of demands that had already been on the public agenda for some years (Wainerman et al., 2008). The demands expressed by various social movements, representing different ideological backgrounds^7^, found the necessary echo in the mass media to raise issues such as the increase in cases of sexual abuse^8^, teenage pregnancy, clandestine abortions, maternal mortality, sexual violence, the rise in the number of people infected with HIV-AIDS or other sexually transmitted infections, as well as the advancement of the age of sexual debut.

However, beyond this support to the bill in Congress, once the law has been sanctioned, the controversy moved to the realm of the media and had as its main axes the contents and modalities of its enforcement. The debate about who should provide this education, what should be the content to be taught or the school period from which these matters should begin to be addressed brought to light dissimilar conceptions not only—nor necessarily—about sexuality, but also about the formal education system. In addition, this debate highlighted the great influence that the Catholic Church exerts not only on civil society, but also on political decisions (Felitti, 2011).

The presentation of sex education as a right^9^ in the curricular guidelines gave the State a leading role in dealing with the subject, a fact that challenged the limits between the public and the private, mainly when it came to those related to students' education. The then Archbishop of La Plata, Héctor Águer, became the main spokesman for the Catholic Church and criticized the law for violating parental authority by relegating the family to a secondary position in the inculcation of values in children^10^ (Esquivel, 2013).

The other main reason of attack on the law by the Catholic Church was the gender perspective that the proponents of the measure intended to imprint on it (Morgade et al., 2018). These criticisms of the law led to a negotiation whose result was the introduction, in the guidelines, of a notion of gender that entails a revision of stereotypes about the masculine and the feminine (Faur, 2020), thus maintaining a binary character in its conceptualization, but incorporating elements that exceed the strictly biological facets in its characterization.

In addition, in this first stage of the enforcement of ESI, we observe a considerable distance from the content of the law in relation to its comprehensive nature and its transversal application^11^, two of the fundamental aspects that differentiate this policy from previous experiences in sex education (Faur, 2012). At a first moment in the upholding of the law, the focus was put on the “evils” that sexual practice can cause, and therefore a “medicalized” view of bodies was brought to the foreground. Such view conceives of bodies as objects of care and prevention. As a result, a biological approach predominated at the time of addressing the contents (Felitti, 2011), which brought with it the persistence of patriarchal and heteronormative values (Lopes Louro, 2018), while the responsibility of teaching them fell on Biology or Natural Sciences teachers or on external specialists in the area of health. This fact is also evident in the materials produced by the Ministry of Education during this period (Dvoskin, 2016).

We can classify this first stage of ESI as a “(scientific) education for the prevention of the consequences of sexuality” (Wainerman et al., 2008). This approach declares as its main objective the prevention and promotion of adolescents' psychophysical healthcare, and therefore restricts its scope to purely medical aspects of control and potentially risky practices. This program is articulated from the premise, more or less explicitly present, that there exist authorized disciplines to address these matters, so it should be the “specialists” who outline the content to be taught and who ought to be consulted. Their status is acquired by the scientific character of their knowledge, framed in a positivist paradigm (Giroux, 2015), so the teaching is done within a hierarchical expert-layperson scheme (doctor-teacher/patient-student).

The outlook on health from which this perspective starts is strongly associated, in its negative sense, with risk, and, in its positive sense, with physiological and psychological well-being, therefore, it is a matter of avoiding potentially morbid situations or conditions:

In more or less subtle ways, care is prescribed “as a couple,” in which stability is equated with “seriousness” and the latter with responsibility [.]. The exercise of sexuality ends up being normatively inserted in an “adequate” scenario (Wainerman et al., 2008, p. 62).

The notion of responsibility is not expressed in moral terms, but it is employed with a preventive sense (either of unwanted pregnancies or of sexually transmitted infections), which is why the main audience to which these recommendations are directed is made up of female adolescents.

The information provided by this program is fundamentally oriented to raising awareness of the risks that sexual practice entails, while other reflexivity elements, such as desire, self-determination or will, are not part of the central axes of this “responsible” sexuality based on “accurate” information. Many of the values and precepts of Christian morality remain in this approach, such as the stable relationship, in which both members are mutually committed and heterosexual.

### The Mobilization of Comprehensive Sex Education

The massive mobilization that took place on June 3, 2015 under the slogan “Ni Una Menos” in different cities of Argentina and that had as its epicenter the National Congress Square, in the Autonomous City of Buenos Aires, revealed the sensitivity of a large part of society to issues connected to gender and sexuality. This awareness was the result of years of activism by feminist and LGBTQA movements, one of the most notorious impacts of which is the “Encuentros Nacionales de Mujeres” (National Women's Forums), which, although held annually since 1987, they have become, since 2010, more relevant for both their large attendance and the dissemination of the activities and debates that take place there (Felitti and Prieto, 2018).

In addition, society's heightened awareness of gender and sexuality matters is also due to the sanction of a series of laws during the governments of Néstor Kirchner (2003–2007) and Cristina Fernández de Kirchner (2007–2011 and 2011–2015), which made visible issues that until then had been silenced in the public sphere (Tabbush et al., 2020). We can mention the Same-sex Marriage Law, enacted in 2010, and the Gender Identity Law, passed in 2012, which deepened the path that the adoption of ESI had already begun in 2006. The Same-sex Marriage Law and the Gender Identity Law employed ESI as a source of support and legitimacy, and they both built on the contents of ESI and the approaches with which to address such contents (Morgade and Fainsod, 2019).

The great impact of the “Ni Una Menos” march in 2015, which brought together a variety of social movements and actors, managed to introduce a series of topics that were not present on the public agenda, especially those related to male violence, in its physical, economic and symbolic dimensions, and also to other matters linked to the pleasure entailed in sexual practices and the freedom to decide on one's own body and identity^12^ The circulation of new topics brought with it the use of new signs with which to address them, such as “forms of sexual dissidence,” “femicide,” “gender violence” or “inclusive language,” as well as the staging of voices of social actors who were absent prior to this conjuncture or whose presence was restricted to very specific realms. In fact, the leaders of feminist and academic movements with a long history in the study of these topics became more important. The field of Social Sciences and Humanities managed to enter the public debate thanks to the criticism of the “patriarchal” biomedical paradigm (Maffía, 2016), the historicization of gender issues (Barrancos, 2017; Segato, 2018) or the employment of non-sexist language (Kalinowski and Sarlo, 2019), among the most outstanding themes. As a result, although the triggering axis of the movement and the march were the feminicides, the treatment of these issues in new spaces and by innovative speakers enabled the circulation of representations that not only put the focus on women's bodies as objects of care, but also on their emancipatory character (Morcillo and Felitti, 2017).

Moreover, the new conjuncture inaugurated by the “Ni Una Menos” march evidenced the irregularities that were unfolding in relation to the implementation of sex education in different institutions of the country. In fact, it highlighted the difficulties entailed in enforcing the law, which reflects a complex interweaving of public policies, institutional policies and social movements (Morgade and Fainsod, 2019).

The criticism aimed at the enforcement of the ESI law brought to the fore, in the first place, the voice of teachers, who in the initial stage had remained hidden behind external specialists coming from the health sector. Although the law stipulated that new issues related to sexuality should be addressed and new perspectives to deal with the issues should be introduced in the classroom, teachers denounced their lack of training to do so and the few pedagogical tools with which the training courses they did provided them (Felitti, 2011). In fact, it was only in 2015 that a subject on this topic was included in the programs of tertiary teacher training institutes, while in Universities—another main source of teacher training in Argentina—this topic still does not enjoy a formal status in their degree programs (Morgade, 2017).

Furthermore, the new agenda established by the feminist movements enabled the emergence of a new social actor, which until then had not been in the spotlight: young people and adolescents. Not only did they play a leading role in the “Ni Una Menos” march and in the “Encuentros Nacionales de Mujeres” (National Women's Forums) (Bidaseca, 2015), but they also voiced their own criticism of ESI enforcement by including this matter among their demands in student centers, both at the secondary and University levels (Dvoskin, 2002), while they even became speakers by producing their own materials on the subject, which were widely distributed in schools^13^. Their first-person narrative marked a sharp contrast with the materials produced by the Ministry of Education during the first stage of the law enforcement, in which students appear predominantly as patients of the action performed by another actor—usually their teacher or their parents—which constitutes students as an object of care, i.e., a passive role that silences their opinion on these topics (Dvoskin, 2016).

In addition to the “Ni Una Menos” march, 2015 was a turning point in Argentina in terms of gender and sexuality, also because of the change in the political party that won the November presidential elections, after which Mauricio Macri became president of the country. The departure of Cristina Fernández de Kirchner from the presidency put an end to 12 years of governments characterized by the extension of people's rights in this area (Tabbush et al., 2020). The government that came to power with Macri as president in December 2015 gave way to a neoliberal model that had a strong impact on the field of education and, in particular, on the enforcement of comprehensive sex education due to the lack of funding for teacher training programs^14^.

This type of policy was characteristic of the Macri government, which gave primacy to a series of discourses that had been relegated during the previous stage (Flax, in press). Specifically in relation to ESI, a resistance movement to this law appeared with great force in Argentina, which had also been developing in Paraguay, Colombia and Brazil: “Con Mis Hijos No Te Metas” (CMHNTM^15^ by its Spanish initials–Do Not Mess With My Children). (Faur, 2020) points out that this movement's opposition to the enforcement of ESI took two forms. On the one hand, in the cultural field, it urged the mobilization of citizens and disseminated messages through social media, whose main target of attack was the “gender ideology,”^16^ a concept that is framed within a biology-oriented paradigm (Dvoskin and Estivalet, 2020). On the other hand, at the institutional level, it developed strategies to prevent the teaching of ESI in schools, especially evidenced in the campaign “No autorizo” (I do not authorize), which consisted in sending parents a model letter that argues for the fact that ESI law is illegal, and deploys arguments that refer to the Argentine national constitution. We see here that the opposition to ESI, in this second moment, moves away from the religious discourse and takes up a legal and scientific perspective.

In the next section, we will present the analysis of the parliamentary debate on the IVE bill, in the Lower House of Congress, and we will present an account of the representations on sex education that were put into circulation there.

## Results

The debate on the IVE bill in the Lower House of Congress began in the morning of June 13, 2018 and ended the following morning, with the result of the half-sanction. A total of 256 deputies expressed their position on the issue, out of which only one abstained from voting. A striking feature of the vote dynamics was that it broke with political party logic: except for the “Frente de Izquierda” (Left Front) bloc that voted entirely in favor of sanctioning the bill, the rest of the party blocks voted both in favor and against.

The social relevance of this problem and the fact that the debate was made public to a mass audience gave way to extremely elaborated and well-argued presentations. This evidences how much consideration the deputies gave not only to the direct addressee of their presentations^17^, i.e., the rest of the participants present in the parliamentary chamber, but also to the indirect addressee, the ordinary citizen who followed the debate through some means of communication and whom the deputies are tasked with representing (Pérez, 2005). As we mentioned in Section Materials and Methods, our analysis of the debate privileged the qualitative dimension, since what interests us is to give an account of the different discourses that circulated on sex education, regardless of the degree of predominance that each of them had.

In the following sections, we will present the representations that circulated about sex education in the debate on IVE. We will begin by outlining the representations generated in the presentations of the deputies who voted against the IVE bill, and then, we will do the same with the presentations of those who were in favor.

### Sex Education Against IVE

The main argument advanced by the deputies who opposed the IVE bill is based on the idea that human life begins with fertilization:

1. Si vamos a dar ese debate de buena fe, hay dos cuestiones liminares que no podemos obviar, que son neurálgicas y constituyen el punto central de todo esto. La primera es a partir de cuándo hay vida, y la segunda, a partir de cuándo existe una persona, un sujeto de derecho. La primera cuestión sólo puede ser respondida desde la ciencia, que hoy nos dice de manera indudable que desde la fecundación existe una vida, un individuo, una vida diferente a la de la madre, con su propia carga genética y su propia secuencia de ADN. Después viene el ordenamiento legal, reconocer que, en esa vida en gestación, en ese sujeto por nacer, hay una persona humana, y el Estado le debe reconocimiento y protección a sus derechos. Es lo que dice el artículo 19 del Código Civil y Comercial (Sr. Incicco: 157)^18^.

If we are going to have this debate in good faith, there are two liminal issues that we cannot ignore, which are neuralgic and constitute the central point of all this. The first one pertains to the moment when there is life, and the second one is connected to the moment when there is a person, i.e., a subject of rights. The first question can only be answered from science, which today tells us in an unquestionable way that, since fertilization, there is a life, an individual, a life different from that of the mother, with its own genetic load and its own DNA sequence. Then comes the legal system, to recognize that, in that life in gestation, in that subject to be born, there is a human person, and the State owes him/her recognition and protection of his/her rights. This is what Article 19 of the Civil and Commercial Code states (Mr. Incicco: 157).

As we observe in the first example taken from the corpus, this argument is supported by two institutions that have a great influence on the behavior of Argentine society: science and law. The appeal to these institutions by Deputy Incicco grants credibility and legitimacy to his words, a discursive strategy that, in turn, places scientific and legal discourses in a position of authority to address the issue of abortion.

From this position, science is assimilated into the positivist paradigm, which presents data as natural phenomena, devoid of any relation to the social conditions in which they take place (Habermas, 1982):

2. Desde el momento de la concepción hay un nuevo ADN separado del de los padres, hay un nuevo ser humano, único y concreto. Este es un hecho biológico y no una opinión subjetiva. Desconocerlo, agregando motivos culturales, es negar un hecho científico (Sr. Zamarbide: 138).

Since the moment of conception there is a new DNA separated from that of the parents, there is a new human being, unique and concrete. This is a biological fact and not a subjective opinion. To ignore it, adding cultural reasons, is to deny a scientific fact (Mr. Zamarbide: 138).

Science is thus configured as a true, objective and transparent discourse, in opposition to ideology, which is associated with subjectivity and totalitarianism:

3. Está claro que legalizar el aborto no debió plantearse como una cuestión ideológica ni política ni de derecha o de izquierda ni mucho menos subjetiva. Tampoco necesitamos en este debate a los guardianes ideológicos, que siempre están y que no soportan a quienes piensan distinto (Sra. Vigo: 175-176).

It is clear that legalizing abortion should not have been raised as an ideological or political question, neither from the right nor from the left, much less subjective. Nor do we need in this debate the ideological guardians, who are always there and who cannot stand those who think differently (Mrs. Vigo: 175–176).

This conception of human life closes off any possibility of debate on the legalization of abortion by implying that it is a form of murder. However, the practice exists in Argentina and entails the main cause of maternal mortality^19^, so this argument is insufficient to silence the debate. This demanded that those who opposed the IVE bill also put forward less extreme arguments, which would allow them to lay the groundwork for discussion of the issue (Angenot, 2015).

Consequently, beyond the condemnatory aspect that the practice of voluntary abortion encompasses, a second argument that we found in this group of deputies was that abortion, whether clandestine or legal, is a traumatic and distressing experience for the person who goes through it, so its legalization cannot constitute a solution to a health problem, but on the contrary, it would function as an aggravating factor. This representation attributes to the sign “abortion” an inherently negative evaluation, so that it is presented as a practice that no person wants to experience:

4. Es innegable que todas las mujeres, sin distinción de clase social, sufren el aborto. Nadie celebra un aborto. Entonces, ¿por qué hablamos de abortar y no de educar (Sra. Hummel: 240).

It is undeniable that all women, without distinction of social class, suffer from abortion. No one celebrates an abortion. Then, why do we talk about abortion and not about education? (Mrs. Hummel: 240).

5. El aborto –digámoslo con todas las letras– es el fracaso de nuestra política pública en prevención y educación sexual (Sra. Scaglia: 178).

Abortion—let us say it in full—is the failure of our public policy on prevention and sex education (Mrs. Scaglia: 178).

In both examples 4 and 5, the intrinsically negative nature of the practice of abortion is presented as presupposed due to its association with suffering or failure. The use of the verb “ser” (to be) in the present tense in the indicative mood gives both assertions a value of timeless truth (Lavandera, 1985), and therefore they are exempt from any kind of questioning, which places their speakers in a position of knowledge. From this perspective, abortion is not a contingent problem inasmuch as its problematic nature does not lie in the socio-historical conditions in which it is performed or in the characteristics of the woman who goes through it.

In fact, the focus of the discussion on how to guarantee the conditions for a safe abortion is shifted, which, from this logic, would constitute an oxymoron, given that what is a cause of suffering cannot be safe. Instead, the question of how to avoid reaching that situation is posed, regarding which education appears as the main tool:

6. La educación integral es la forma más sincera de ocuparse de las problemáticas existentes en nuestra sociedad, es una forma concreta y real de prevenir situaciones que afectan a los más vulnerables y de construir una sociedad igualitaria, donde la eliminación de una vida no sea una práctica lamentablemente aceptada (Sr. Olivares: 103).

Comprehensive education is the sincerest way to deal with the problems that exist in our society, it is a concrete and real way to prevent situations that affect those who are the most vulnerable and to build an egalitarian society, where the elimination of a life is not a regrettably accepted practice (Mr. Olivares: 103).

More or less explicitly, in examples 4, 5, and 6, formal education is presented as a method to prevent unwanted situations, such as a pregnancy. The solution to the problems generated by the practice of clandestine abortion lies mainly, from this argumentative logic, in the correct enforcement of the ESI law:

7. Estamos fallando como legisladores, porque no estamos controlando la aplicación de las leyes de salud y educación sexual integral (Sra. Rosso: 106).

We are failing as legislators, because we are not controlling the enforcement of health and comprehensive sex education laws (Ms. Rosso: 106).

8. Tenemos que comprometernos todos a trabajar en la implementación de la ley de educación sexual en aquellas escuelas donde aún no se imparten los contenidos obligatorios y capacitar a nuestros docentes para que dicten estas temáticas en las aulas (Sr. Arce: 218).

We must all commit ourselves to working on the enforcement of the law on sex education in those schools where the compulsory content is not yet taught and to train our teachers to teach these topics in the classrooms (Mr. Arce: 218).

The use of first-person plural subjects (which, in Spanish, are elided) by the speakers in examples seven and eight groups together all the legislators in the same collective, regardless of their respective position on the ESI law. The exhortation to the rest of the legislators to enforce an already sanctioned law characterizes this actor as one of the culprits of the poor implementation of sex education in Argentina and, therefore, insinuates that they are responsible for the occurrence of clandestine abortions. At the same time, legislators are given a leading role in reversing this situation, since the correct upholding of the law would generate the conditions so that these practices—and their consequences—do not continue to occur:

9. Una forma de protección debe partir de la educación sexual y de los diversos mecanismos y medios para incorporar conocimientos y valores sobre los procesos de concepción y embarazo. Sin duda, es responsabilidad del Estado asumir este rol protector, poniendo al alcance de todas las mujeres distintas herramientas que garanticen su protección (Sr. Baldassi: 207).

A form of protection must start with sex education and the various mechanisms and means to incorporate knowledge and values about the processes of conception and pregnancy. Without a doubt, it is the responsibility of the State to assume this protective role, making available to all women different tools that guarantee their protection (Mr. Baldassi: 207).

10. Morimos porque no recibimos una verdadera educación sexual integral (Sra. Bianchi: 70).

We die because we don't receive truly comprehensive sex education (Ms. Bianchi: 70).

Sex education is presented as a realm for the provision of information to students, who must internalize this knowledge in order not to experience the suffering or condemnation that the practice of abortion entails. Thus, it is implied that unwanted pregnancy is a product of the ignorance of adolescents. In addition, women are configured as the main addressee of this measure and are constituted as a subject of care, who must be protected.

From this position, the predominant function of sex education is to prevent the unwanted consequences of sexual practice (such as abortion or sexually transmitted infections), so that the issue of sexuality is reduced to its biological content. In this way, the representation of sex education as an argument against the IVE bill configures the scientific discourse—specifically, the biomedical one—as the only one that is legitimized to address this problem. Sexuality becomes a risk practice and education appears as a method of prevention and protection.

### Sex Education in Favor of Abortion

The debate on IVE reached Congress after the bill had been rejected seven times, so its discussion in the parliamentary chamber was presented as an achievement by those who voted in favor:

11. Por primera vez en 35 años de democracia este Congreso trata la legalización del aborto. Esto tiene que ver con una lucha que un colectivo de mujeres comenzó hace muchos años bajo el lema “aborto legal, educación sexual y ley de procreación responsable” (Sra. Ocaña: 172).

For the first time in 35 years of democracy, this Congress deals with the legalization of abortion. This has to do with a struggle that a group of women began many years ago under the slogan “legal abortion, sex education and responsible procreation law” (Ms. Ocaña: 172).

12. Las mujeres nos hemos convertido en impulsoras y protagonistas de un proceso de resistencia. El feminismo es una respuesta política organizada, popular y diversa que se ha construido colectivamente. Es una revolución, porque implica un completo cambio de paradigma. Esto no es casual, ya que somos hijas y nietas de las Madres y de las Abuelas de Plaza de Mayo […]. Somos también las que aprendimos de las mujeres piqueteras, que fueron aquellas primeras mujeres que salieron a resistir el ajuste y la privatización cuando sus maridos se quedaban sin trabajo. Al mismo tiempo, somos las compañeras de Cristina Fernández de Kirchner, que se atrevió a cometer un pecado mortal: ser mujer y enfrentarse al poder. Eso le valió un embate machista y misógino contra su persona sin antecedentes, salvo el de la querida Evita (Sra. Volnovich: 188).

We, women, have become the promoters and protagonists of a process of resistance. Feminism is an organized, popular and diverse political response that has been built collectively. It is a revolution, because it implies a complete paradigm shift. This is not accidental, since we are the daughters and granddaughters of the Mothers and Grandmothers of *Plaza de Mayo*^20^ [.]. We are also the ones who learned from the *piquetera*^21^ women, who were the first women to go out and resist adjustment and privatization when their husbands were out of work. At the same time, we are the companions of Cristina Fernandez de Kirchner^22^, who dared to commit a mortal sin: to be a woman and to confront power. That earned her a *machista* and misogynist attack against her person with no background except that of our beloved Evita^23^ (Mrs. Volnovich: 188).

As shown in examples 11 and 12, this achievement is attributed primarily to women. The reference to the feminist movements, to the Mothers and Grandmothers of Plaza de Mayo, to the women *piqueteras* and to leaders such as Eva Duarte de Perón and Cristina Fernández de Kirchner, characterizes women as a social actor who is the protagonist of the social changes produced, so that their voice holds a value of differential legitimacy in the debate. Thus, the legalization of abortion is presented as the conquest of a new right, a characteristic that relates it to the ESI law:

13. En el 2006 hemos sancionado la ley de educación sexual integral, que aún hoy continúa con dificultades para su aplicación por los mismos sectores retrógrados y conservadores, que se niegan a garantizar el derecho que hoy estamos debatiendo (Sra. Mendoza: 51).

In 2006 we passed the law on comprehensive sex education, which even today continues to face difficulties in its enforcement caused by the same backward and conservative sectors that refuse to guarantee the right we are debating today (Ms. Mendoza: 51).

14. He escuchado que algunos diputados proponen educar, como si se pudiera reemplazar a la política con pedagogía. Es nuestra primera consigna. Por eso impulsamos desde el primer día la educación sexual integral en los colegios, porque sabemos que previenen los embarazos no deseados. Pero si bien es ley desde octubre de 2006, también sabemos que es una norma que no se cumple […]. Y la verdad, para que la educación tenga sentido, hace falta que las mujeres estemos vivas (Sra. Álvarez Rodríguez: 204).

I have heard some Members of Parliament propose education, as if politics could be replaced by pedagogy. This is our first slogan. That is why we have been promoting comprehensive sex education in schools from day one, because we know that it prevents unwanted pregnancies. But even though it has been a law since October 2006, we also know that it is a rule that is not followed [.]. And the truth is that for education to make sense, women must be alive (Ms. Alvarez Rodriguez: 204).

Although the position shown in example 14 shares the representation of sex education as a method for preventing unwanted pregnancies, which we observed in the previous section, education is not presented as a measure that can reverse, at least in the short run, the problem of abortion. The death of women due to clandestine abortions represents an urgency that, from this argumentative logic, sex education cannot address, making the latter a necessary but insufficient condition to combat the former.

Moreover, the ineffective enforcement of the law is not attributed to the group of legislators as a whole, as the deputies opposed to IVE maintained, but only to a sector of such group: precisely those who oppose the sanction of IVE. At the same time, the cut in the budget produced by the Macri government is mentioned as another fundamental factor that makes the correct upholding of this ruling impossible:

15. Los recursos destinados a la educación sexual integral han disminuido en trece mil millones de pesos, los fondos destinados a las capacitaciones son menores porque de cincuenta y cinco mil docentes capacitados en 2015 pasamos lamentablemente a mil cincuenta (Sra. Siley: 192).

The resources allocated to comprehensive sex education have been reduced by 13 billion pesos, the funds allocated to training are less because from 55,000 teachers trained in 2015, we have unfortunately gone down to 1,050 (Ms. Siley: 192).

Moreover, in the presentations of the deputies who voted in favor of IVE, sex education does not have the sole function of making information of a preventive nature available:

16. Espero que se desarrolle la educación sexual integral con la capacidad de oír y de habilitar escenarios que permitan lograr un verdadero diálogo que dé a las pibas y los pibes herramientas suficientes. Además, se requiere una educación integral para que puedan valorarse como personas, como mujeres en la construcción de su género, con el respeto a la identidad autopercibida, otro derecho ganado en esta democracia (Sra. Horne: 168).

I hope that comprehensive sex education will be developed with the capacity to listen and to set up scenarios that will make it possible to achieve a true dialogue that gives young women and men sufficient tools. Furthermore, comprehensive education is required so that they can value themselves as persons, as women in the construction of their gender, showing respect for their self-perceived identity, another right earned in this democracy (Ms. Horne: 168).

17. En esa tarea de todes está la responsabilidad del Estado, de la planificación familiar de cada uno de los proyectos de vida de hombres y mujeres, de mujeres gestantes, de poder llevar adelante, en el momento que quieran, la planificación de su familia con educación sexual y teniendo a disposición todos los medios para llevar adelante la salud reproductiva como lo dispongan […]. Además de la batalla cultural estaríamos avanzando en dar la posibilidad a la mujer y a la mujer gestante que quiera practicar […] la interrupción voluntaria de su embarazo si no fue planificado o deseado. Esto tiene un complemento que ya está sancionado por ley, gracias a la batalla cultural que llevó adelante el movimiento feminista, que es la educación sexual con una mirada federal e integral (Sr. Cleri: 220).

In this task of all^24^ it is the responsibility of the State, of the family planning of each one of the life projects of men and women, of pregnant women, to be able to carry out, at the moment that they want, the planning of their family by having access to sex education and having available all the means to enjoy their reproductive health as they see best […]. In addition to fighting a cultural battle, we would be making progress in giving women and pregnant women who want to do it [.] the voluntary interruption of their pregnancy if it was not planned or desired. This has a complement that is already sanctioned by law, thanks to the cultural battle fought by the feminist movement, which is sex education with a federal and comprehensive approach (Mr. Cleri: 220).

In example 16, we observe that the idea of dialogue appears in relation to sex education, a notion that opens the possibility for the circulation of not only teachers' voice but also that of the students (Freire, 1969). In addition, in both examples, sex education is related to identity, desire, and the possibility of making decisions, a concept that does not restrict it to a public health issue and, therefore, sexuality is not reduced to the unwanted consequences of sexual practice. Both statements are formulated from a notion of volition, a fact evidenced in the verb “I hope” in example 16 or in the conditional nuance of example 17, a modality that does not express what it is, but what could be (Palmer, 1986). Therefore, the speakers do not place themselves in a position of knowledge or authority, but rather establish a dialogical opening toward other voices and alternative meanings.

This representation of sex education enables an evaluation of the sign “abortion” that does not presuppose its negative character, but presents this practice as constitutive of people's sexual life:

18. La ciencia tampoco explica el aborto. Ninguna de las ciencias lo explica; ni la biología, ni la medicina, ni la sociología, ninguna. Solamente sabemos que hay algunas mujeres que quieren tener familia y otras mujeres que no quieren tenerla. Y cuando uno pregunta cuál es la razón, la razón aparece como un deseo: deseo de ser madre, deseo de tener un hijo, deseo de querer, deseo de amar (Sr. Del Cerro: 241).

Nor does science explain abortion. None of the sciences explains it; neither biology, nor medicine, nor sociology, none. We only know that there are some women who want to have a family, and other women who do not want to have a family. And when one asks what the reason is, the reason appears as a desire: a desire to be a mother, a desire to have a child, a desire to want, a desire to love (Mr. Del Cerro: 241).

The introduction of desire as a criterion for deciding in place of reason implies a cultural paradigm shift. Consequently, scientific discourse is displaced from the position as the sole authority for addressing the issue of abortion.

Although prevention remains one of the values associated with the sign “sex education” in the presentations given by the deputies who expressed themselves in favor of the IVE bill, the function of this measure is not reduced exclusively to it. The incorporation of other contents, such as desire, identity, or the possibility of making decisions, gives legitimacy to other social actors to pronounce themselves on the subject beyond health specialists.

Rationality is no longer the only parameter to determine who are the authorized speakers to address these issues, a fact that implies a cultural change with respect to the hegemonic discourses that prevailed in modern Western societies (Giroux, 1997). Consequently, the characterization of women as the protagonists of social struggles in Argentina casts them in a central role in this process.

## Discussion

In the previous section, we characterized the representations on comprehensive sex education that were put into circulation by both the deputies who voted in favor of the IVE bill and those who voted against it. Both presented the enforcement of ESI as an argument to support their positions, a paradox that makes it clear that there is a dispute over the meaning attributed to this measure.

Thus, we have showed that the deputies against IVE conceive of sex education exclusively as a method for preventing the unwanted consequences of sexual practice. This representation reduces sexuality to its biological aspects, so that the biomedical discourse is the only one that is authorized to address this problem. Education is then presented as the spread of knowledge (neutral, transparent, and objective) from a specialist-speaker to an ignorant-student who must internalize this knowledge in order to become aware of the risks that sexual practice entails.

The deputies in favor of IVE, in contrast, ascribe other values to the sign “comprehensive sex education.” The presentation of this measure as a right allows for the incorporation of such themes as identity, desire and the possibility of choosing, i.e., contents that bring to the fore the voice not only of health specialists, but also of young people and women, both protagonists in the new situation established by the “Ni Una Menos” march in 2015.

However, we have observed that some values are shared by both positions. The preventive nature of the measure is asserted by both groups, a dimension that highlights the biomedical paradigm that frames the debate on these issues and maintains its initiative in the valuing of the sign “science”:

19. Nosotros, que sí luchamos por la educación sexual, laica y científica mientras acá algunos acaban de descubrirla, cuando la han obstaculizado (Sra. Del Plá: 82).

We, who do fight for sexual, lay, and scientific education, while here there are some who have just discovered it, even when they have only hindered its provision (Mrs. Del Plá: 82).

20. Si no tenemos en cuenta a la ciencia, quedaremos atrapados en una política ideológica, de descarte del ser humano (Sra. Martínez Villada: 115).

If we do not take science into account, we will be trapped in ideological politics of discarding the human being (Ms. Martínez Villada: 115).

This evaluation of the sign “science” as objective, transparent and neutral is corroborated in the contrast that is made with respect to the pejorative value ascribed to the sign “ideology,” a value that appears both in the presentations of the deputies opposing IVE, as evidenced in example 20, and in those of the deputies who voted in favor, as is observed in the following example:

21. Estamos tratando un tema de salud pública que no puede ser abordado con anteojeras ni morales ni éticas ni ideológicas ni, mucho menos, religiosas (Sra. Mendoza: 51).

We are dealing with a public health matter that cannot be addressed with moral or ethical or ideological blinders, much less religious ones (Ms. Mendoza: 51).

The concept of ideology as false consciousness (Marx and Engels, 1932), which appears in example 21, has a pejorative character since it conceives of it as a veil that does not permit “seeing” reality, just as an obstacle to truth. Thus, the representation of scientific discourse as transparent and objective, typical of the positivist paradigm (Giroux, 1997), is reproduced, and, in the field of sex education, such discourse emerges in the guise of biomedical discourse. This discourse puts in the foreground the urgent problems that must be addressed in relation to sexuality, that is, the ills that sexual practice can entail—such as unwanted pregnancies, clandestine abortions, or sexually transmitted infections, but it leaves in oblivion the emancipatory character that this type of education can have if questions related to identity, desire, or freedom of choice are problematized.

In addition, both discourses denounce the failures observed in the enforcement of ESI. However, as we showed in the previous section, the causes of these failures are different for each group of deputies. In fact, those who voted in favor of IVE allocate the opposition deputies the responsibility for having obstructed the implementation of this policy and position such opposition deputies as spokespersons for the Catholic Church:

21. Si algo ha distinguido al oscurantismo de la cúpula de la Iglesia Católica durante todos estos años es oponerse a leyes como la que hoy estamos discutiendo aquí. Por esos motivos, se han opuesto a la independencia nacional, al fin de la esclavitud, a la ley 1.420 –de educación común, laica, gratuita y obligatoria–, a la reforma universitaria, al divorcio, a la educación sexual, al matrimonio igualitario, al voto femenino y ahora al derecho al aborto legal (Sr. Del Caño: 123).

If there is anything that has distinguished the obscurantism of the Catholic Church leadership during all these years, it is its opposition to laws such as the one we are discussing here today. For these reasons, they opposed national independence, the end of slavery, Law 1,420—on common, secular, free and obligatory education -, University reform, divorce, sex education, same-sex marriage, women's suffrage and now the right to legal abortion (Mr. Del Caño: 123).

Although this institution had expressed its opposition to the sanction of ESI, in 2006, through its most representative members—as is the case of the then Archbishop of La Plata, Héctor Aguer—the deputies who voted against IVE, in 2018, not only demanded the enforcement of ESI, but also moved away from the religious discourse to pronounce themselves on the subject. Instead, they appealed to scientific and legal discourses to support their positions:

22. No hay ninguno de los argentinos que quiera producirse un aborto […]. En la Argentina el concepto no pasa por la Iglesia Católica, pasa por la importancia que tiene la vida y, cuando se habla de la vida, no se habla de cualquier cosa (Sr. Ramón: 79).

No Argentine wants to have an abortion [.]. In Argentina the concept has nothing to do with the Catholic Church, but it is connected to the importance that life has, and when we speak about life, we do not just speak about a minor thing (Mr. Ramon: 79).

23. Estos proyectos de ley legalizarían una violación a la Constitución Nacional y a sus tratados internacionales (Sr. Pereyra: 94).

These bills would legalize a violation of the National Constitution and its international treaties (Mr. Pereyra: 94).

Unlike what happened in the first stage of ESI, in which the opposition to the measure was expressed by means of a religious discourse while those who defended it adopted a stance that adhered to a biomedical discourse, in this second moment, the discursive scene has changed. The legal and scientific discourses emerge as the main stances in opposition to IVE, although the deputies who support this bill continue to configure the Catholic Church as their counteraddressee (Verón, 1987), that is, as the actor that must be defeated in order to achieve the legalization of abortion and the correct enforcement of ESI.

Perhaps it is this dialogue of the deaf (Angenot, 2015) that prevents abortion from being a legal practice in Argentina or sex education from being correctly imparted in schools from a gender perspective. Or maybe it is part of an ongoing process that is slowly shaping feminist discourse as an emerging, emancipatory discourse that will remove patriarchal discourse from its hegemonic place.

## Data Availability Statement

The raw data supporting the conclusions of this article will be made available by the authors, without undue reservation.

## Author Contributions

All authors listed have made a substantial, direct and intellectual contribution to the work, and approved it for publication.

## Conflict of Interest

The author declares that the research was conducted in the absence of any commercial or financial relationships that could be construed as a potential conflict of interest.

## Footnotes

^1^IVE stands for “Interrupción Voluntaria del Embarazo” in Spanish. In this article, we have decided to employ the acronym in the original language.

^2^Regarding the name of the march, we have also decided to refer to it in the original language, i.e., “Ni Una Menos.”

^3^To learn about the track record of the National Campaign for the Right to Free, Legal and Safe Abortion and the bill presented, see Available online at: http://www.abortolegal.com.ar/15-anos-de-campana/.

^4^We can include in this set the laws of *Educación Sexual Integral* (Comprehensive Sex Education) (2006), *Protección Integral de las Mujeres* (Comprehensive Protection of Women) (2009), *Matrimonio Igualitario* (Same-sex Marriage) (2010), *Identidad de Género* (Gender Identity) (2012), *Fertilización Asistida* (Assisted Fertilization) (2013) and *Micaela* (2019). For a detailed description of these laws, please visit the website of the Argentine Congress. Available online at: https://www.congreso.gob.ar/leyes.php.

^5^ESI stands for “Educación Sexual Integral” in Spanish. In this article, we have decided to employ the acronym in the original language.

^6^We are aware that feminist discourse is not homogeneous, but rather several strands coexist within it, not necessarily compatible with one another. However, for the purposes of this paper, such heterogeneity is not significant.

^7^Only in order to simplify the presentation, and being aware of the error entailed in considering these ideological formations as homogeneous groups, we can place the different social movements in a *continuum* whose poles would be “Catholic Church” and “Human Rights.” We will simply mention some of these movements and social groupings, which have had more significant social repercussion in Argentina: GLTTB (gays, lesbians, transvestites, transsexuals and bisexuals), the CHA (by its Spanish initials, *Comunidad Homosexual Argentina*) (Argentinean Homosexual Community), MADEL (by its Spanish initials, *Mujeres Autoconvocadas para Decidir en Libertad*) (Self-Convened Women to Decide in Freedom), *Familia y Vida* (Family and Life), *la Marcha del Orgullo* (the Gay Pride Parade), and *Encuentros Nacionales de Mujeres* (National Women's Forums) (Pecheny et al., 2008).

^8^In 2006, two emblematic cases of abuse were reported in the mass media because they involved disabled women who were victims of rape and who were denied authorization by the justice system to have an abortion (see the digital edition of *Página/12*, August 12, 2006).

^9^In the *Lineamientos curriculares para la Educación Sexual Integral* (Curricular Guidelines For Comprehensive Sex Education), the following right is asserted: “people's right to receive information and acquire knowledge, duly validated by the scientific community, with regard to the care, promotion and prevention of risks and damage to one's own health, the respect for one's own body and that of others, the prevention of infectious diseases and all knowledge that contributes to the exercise of a healthy, responsible and full sexuality” (Curricular Guidelines For Comprehensive Sex Education, Ministry of the Nation, Buenos Aires, April 17, 2008, p. 9).

^10^Despite the fact that the law explicitly establishes that the definition of the content on sex education is at the discretion of each educational community (Law 26,150, article 5), whose members include parents.

^11^Law 26.150, *Programa Nacional de Educación Sexual Integral* (National Program of Comprehensive Sex Education, article 1).

^12^The manifesto can be read Available online at: http://niunamenos.org.ar/.

^13^The most emblematic case was a manual produced by students from *Carlos Vergara* High School N°. 14, in the city of La Plata (Province of Buenos Aires), together with their teachers Andrea Beratz and Jesabel Agüero. The material can be downloaded for free. Available online at: https://www.comisionporlamemoria.org/jovenesymemoria/wpcontent/uploads/sites/21/2019/03/donde-esta-mi-esi.pdf.

^14^Báez and Fainsod point out that “while in 2015 the budget [for comprehensive sex education] was 55 million pesos, in 2016 it was reduced to 27 million and, in 2017, it was 43 million, which is still much less than what was allocated in 2015 if inflation is taken into account” (2018, p. 4).

^15^The genesis of this movement can be seen in the documentary *El género bajo ataque*, Available online at: https://www.youtube.com/watch?v=PjJZQGjs9ck.

^16^In Argentina in August of 2018, through social networks, the *CMHNTM* movement disseminated information in a material that had mass circulation. It was called “Ideología de género. Mitos y verdades” (Gender Ideology. Myths and truths), and it can be found at www.conmishijosno.com.

^17^García Negroni (2016) distinguishes between the direct addressee, who shares the interlocution relationship with the speaker and to whom the message is explicitly addressed, and the indirect addressee, who is not part of the interlocution relationship, even when their presence is planned by the speaker, something which usually leaves traces on the text surface.

^18^All the examples were taken from the stenographic version of the session journal of the Lower House of Representatives, Meeting 8 (13/6/18), Available online at: https://www.diputados.gov.ar/secparl/dtaqui/diario_sesiones/index.html. After each fragment, we include, in parentheses, the gender and the name of the deputy responsible for the presentation and the page number of the journal.

^19^It is estimated that around 450 thousand clandestine abortions are performed in Argentina every year and that, since the country's return to democracy in 1983, approximately three thousand women have died due to unsafe abortions (Mario and Pantelides, 2009).

^20^The Mothers of Plaza de Mayo is an Argentine association that was created during the last dictatorship in that country (1976–1983) to recover the disappeared detainees alive. After the dictatorial regime ended, its objective was to establish who were the people responsible for the crimes against humanity and promote their prosecution. On the other hand, the Grandmothers of Plaza de Mayo is an Argentine human rights organization whose purpose is to locate and restore to their legitimate families all the babies and children appropriated by the last military dictatorship, create the conditions to prevent this crime against humanity and obtain the corresponding punishment for all those responsible.

^21^The name “piquetera” comes from the form of protest carried out by certain movements of the unemployed, which consists of the installation of pickets in strategic places, to totally or partially make it impossible to circulate on streets, roads or routes.

^22^Cristina Elisabet Fernández de Kirchner is an Argentine politician. She was President of the Argentine Nation (2007–2015) and currently, she serves as Vice President of the Argentine Nation.

^23^María Eva Duarte (1919–1952), better known as Evita, was an Argentine politician. She was the First Lady of the Argentine Nation during the presidency of her husband Juan Domingo Perón between 1945 and 1952 and president of the Peronist Feminine Party and the Eva Perón Foundation.

^24^In the Spanish original text, there is the use of the pronoun “todes,” instead of the normative “todos” or “todas.” The replacement of the masculine morpheme “o” or the feminine morpheme “a” with the morpheme “e” is in line with a non-binary employment of language, driven by, to a great extent, feminist movements in Argentina.
